# Probing the role of the C_2_F domain of otoferlin

**DOI:** 10.3389/fnmol.2023.1299509

**Published:** 2023-12-12

**Authors:** Han Chen, Qinghua Fang, Fritz Benseler, Nils Brose, Tobias Moser

**Affiliations:** ^1^Institute for Auditory Neuroscience and InnerEarLab, University Medical Center Göttingen, Göttingen, Germany; ^2^Collaborative Research Center 889, University of Göttingen, Göttingen, Germany; ^3^Auditory Neuroscience and Synaptic Nanophysiology Group, Max Planck Institute for Multidisciplinary Sciences, Göttingen, Germany; ^4^Göttingen Graduate Center for Neurosciences, Biophysics and Molecular Biosciences, University of Göttingen, Göttingen, Germany; ^5^Department of Molecular Neurobiology, Max Planck Institute for Multidisciplinary Sciences, Göttingen, Germany; ^6^Multiscale Bioimaging Cluster of Excellence (MBExC), University of Göttingen, Göttingen, Germany

**Keywords:** hearing, cochlea, ribbon synapse, exocytosis, otoferlin, deafness

## Abstract

Afferent synapses of cochlear inner hair cells (IHCs) employ a unique molecular machinery. Otoferlin is a key player in this machinery, and its genetic defects cause human auditory synaptopathy. We employed site-directed mutagenesis in mice to investigate the role of Ca^2+^ binding to the C_2_F domain of otoferlin. Substituting two aspartate residues of the C_2_F top loops, which are thought to coordinate Ca^2+^-ions, by alanines (*Otof^D1841/1842A^*) abolished Ca^2+^-influx-triggered IHC exocytosis and synchronous signaling in the auditory pathway despite substantial expression (~60%) of the mutant otoferlin in the basolateral IHC pole. Ca^2+^ influx of IHCs and their resting membrane capacitance, reflecting IHC size, as well as the number of IHC synapses were maintained. The mutant otoferlin showed a strong apex-to-base abundance gradient in IHCs, suggesting impaired protein targeting. Our results indicate a role of the C_2_F domain in otoferlin targeting and of Ca^2+^ binding by the C_2_F domain for IHC exocytosis and hearing.

## 1 Introduction

The afferent synapses formed by inner hair cells (IHCs) with spiral ganglion neurons (SGNs) indefatigably transmit sound information at high rates with utmost temporal precision (Safieddine et al., 2012; Johnson et al., 2019; Moser, 2020; Rutherford et al., 2021; Moser et al., 2023). Each presynaptic active zone located at the basal IHC pole drives the firing of its postsynaptic SGN up to a hundred spikes per second for strong sound intensities. In order to cope with this daunting task, IHCs seem to employ a specialized molecular machinery that notably involves the presynaptic ribbon, an electron-dense proteinaceous structure tethering synaptic vesicles (SVs; reviewed by Wichmann and Moser, 2015; Moser, 2020). Deciphering the molecular physiology of sound encoding at the afferent IHC synapses is an ongoing effort (Safieddine et al., 2012; Johnson et al., 2019; Moser, 2020; Rutherford et al., 2021). For example, multidomain active zone (AZ) scaffold proteins bassoon (Khimich et al., 2005; Buran et al., 2010), piccolino (Müller et al., 2019; Michanski et al., 2023), RIM (Rab-3-interacting molecule) 2 and RIM3 (Jung et al., 2015a; Picher et al., 2017b), RIM-BP (Krinner et al., 2017, 2021), and the ribbon specific protein RIBEYE (Becker et al., 2018; Jean et al., 2018) were shown to be required for normal sound encoding at the afferent IHC synapses. Surprisingly, the presence and function of the SNARE (soluble N-ethylmaleimide-sensitive-factor attachment receptor)-based neuronal SV fusion machinery (Nouvian et al., 2011; Calvet et al., 2022) and its regulators such as complexins (Strenzke et al., 2009), synaptotagmins (Beurg et al., 2010; Johnson et al., 2010; Reisinger et al., 2011), or Munc13/CAPS (Ca^2+^-dependent activator proteins for secretion) priming factors (Vogl et al., 2015) at IHC synapses are less clear. This suggests a deviation of IHC AZs from the molecular machinery of conventional synapses. In line with this notion, human genetic studies have discovered mutations in a number of deafness-associated genes that cause auditory synaptopathy often without further neurological deficits. The audiological signature of auditory synaptopathy is pathological or absent auditory brainstem responses (ABRs) despite maintained cochlear amplification as evident by otoacoustic emissions or cochlear microphonic potentials (Moser et al., 2006; Santarelli et al., 2015; Moser and Starr, 2016).

**Figure 1:**
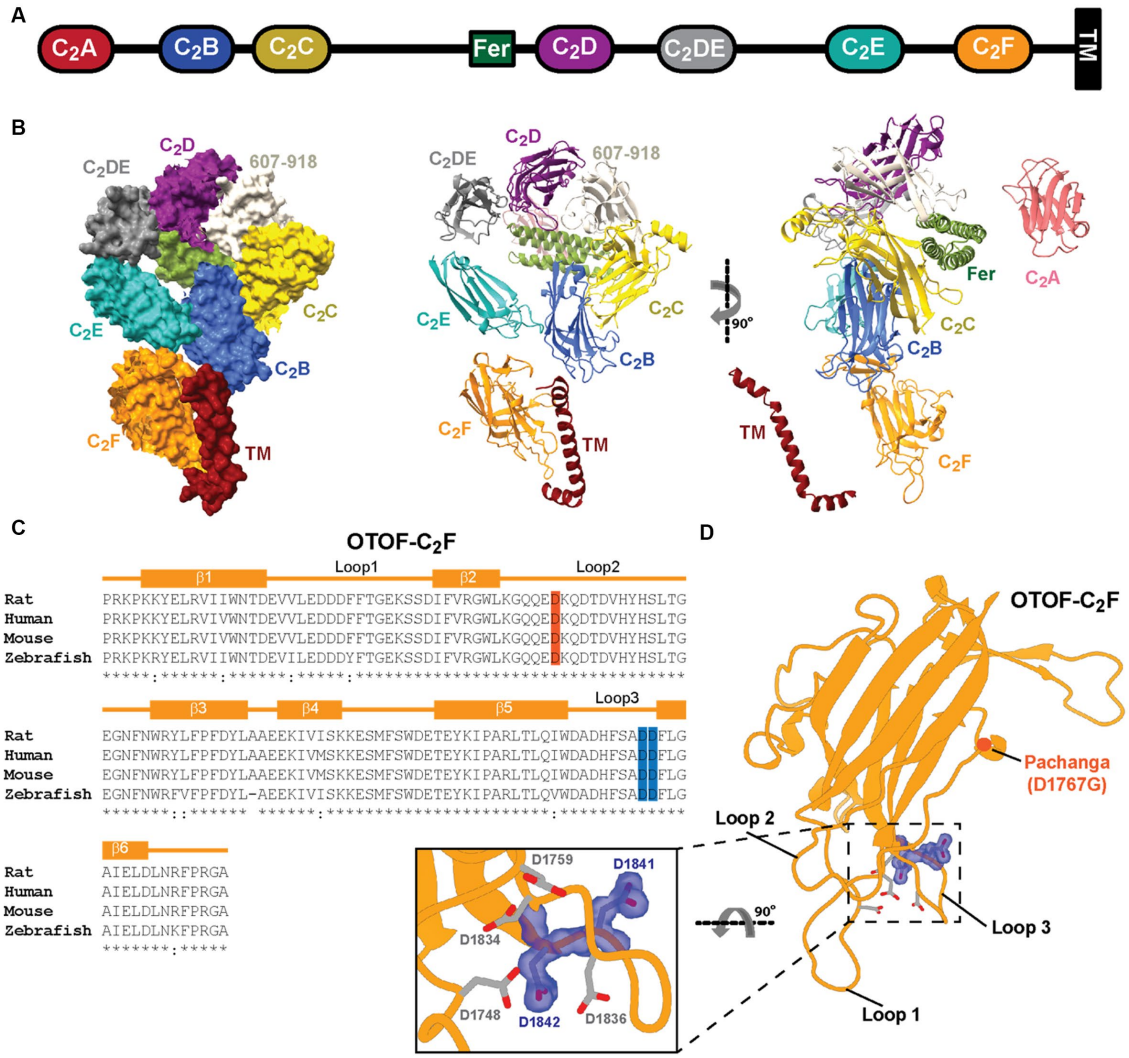
Predicted structure of otoferlin and amino acids of C_2_F targeted by mutagenesis. **(A)** Domain organization of otoferlin. Otoferlin is a single-pass transmembrane protein (1,997 amino acids in humans) consisting of seven C_2_ domains (C_2_A-F and C_2_DE), one C_2_ domain-like domain (amino acids 607–918, not displayed for simplicity), one FerB domain and a C-terminal transmembrane ™ domain. **(B)** AlphaFold2 predicted the overall structure of the otoferlin protein in surface representation and ribbon diagram. **(C)** Structure-based sequence alignment of otoferlin C_2_F domain among species: aspartates substituted by alanines (D1841/1842A) are highlighted in blue. **(D)** Predicted model of otoferlin C_2_F domain. The putative Ca^2+^ binding pocket is formed by the top loops (loop1 and loop3). Potential Ca^2+^ binding aspartates are shown in sticks mode, the targeted aspartates in purple, and the *Pachanga* mutation, D1767G, is highlighted in red.

Genetic auditory synaptopathy can involve deficiency or dysfunction of the Ca_V_1.3 Ca^2+^ channel (Baig et al., 2011) or its modulators CaBP2 (Schrauwen et al., 2012; Picher et al., 2017a), vesicular glutamate transporter 3 (Greene et al., 2001), and otoferlin (Yasunaga et al., 1999; Roux et al., 2006). Otoferlin, a tail-anchored (Vogl et al., 2016) multi-C_2_-domain protein specific to hair cells (Roux et al., 2006; Figure 1A), is a member of the ferlin protein family involved in membrane trafficking and repair that are of major disease relevance (McNeil and Kirchhausen, 2005; Lek et al., 2012; Pangršič et al., 2012). Otoferlin is distributed broadly within IHCs (Roux et al., 2006; Pangrsic et al., 2010; Revelo et al., 2014; Strenzke et al., 2016), likely reflecting its presynaptic function in the basolateral IHC pole and an additional role in constitutive, non-synaptic membrane trafficking (Revelo et al., 2014). Otoferlin seems to have a multifaceted role in the SV cycle at IHC AZs (Moser and Starr, 2016), serving as (i) candidate Ca^2+^ sensor of SV fusion (Roux et al., 2006; Johnson and Chapman, 2010; Michalski et al., 2017) and (ii) promoter of Ca^2+^ dependent SV replenishment at the release sites (Pangrsic et al., 2010; Strenzke et al., 2016; Vogl et al., 2016; Michalski et al., 2017; Tertrais et al., 2019) likely via SV tethering (Vogl et al., 2015) and mediating exocytosis–endocytosis coupling (Duncker et al., 2013; Jung et al., 2015b; Kroll et al., 2019, 2020; Tertrais et al., 2019).

Here, we used a multidisciplinary approach to decipher the function of the most C-terminal C_2_ domain (C_2_F) of otoferlin that appears to be critical for otoferlin function as assessed by virus-mediated rescue of IHC exocytosis (Tertrais et al., 2019). Human subjects with missense *OTOF* mutations affecting the C_2_F domain exhibit auditory synaptopathy with or without temperature dependence (Migliosi et al., 2002; Rodríguez-Ballesteros et al., 2008; Marlin et al., 2010; Iwasa et al., 2013; Vona et al., 2020). Previous mouse mutagenesis analysis of C_2_F employed the N-ethyl-N-nitrosourea (ENU)-generated *Pachanga* mouse (*Otof^D1767G/D1767G^*) substituting an aspartate of a bottom loop by a glycine (Schwander et al., 2007) and revealed a lack of ABRs despite otoacoustic emissions being present. An in-depth analysis of *Otof^D1767G/D1767G^* IHCs showed otoferlin levels to be reduced to approximately 25% of *Otof^+/+^* IHCs. *Otof^D1767G/D1767G^* IHCs retained Ca^2+^ triggered SV fusion and transmitter release but showed impaired SV replenishment, which resulted in low rates and fatigue of synaptic transmission and sound-evoked firing (Pangrsic et al., 2010; Takago et al., 2019). To disrupt Ca^2+^ binding to the C_2_F domain in IHCs, we substituted aspartate residues (D) within the top loops that putatively coordinate Ca^2+^ ions by alanine (A) residues in CRISPR/Cas9 generated knock-in mice. The novel mouse mutant lacks auditory brainstem responses (ABRs) and Ca^2+^ influx-triggered IHC exocytosis. Basolateral otoferlin levels were reduced in IHCs. Given the moderate (by 40%) reduction of basolateral otoferlin in *Otof^D1841/1842A^* IHCs, we propose the impaired synaptic sound encoding primarily result from defective Ca^2+^-sensing by the C_2_F domain.

## 2 Materials and methods

### 2.1 Structure prediction

The structure of otoferlin was predicted by AlphaFold 2 (Jumper et al., 2021), which can be directly downloaded in UniProt (Q9HC10, uniprot.org). The protein structure figures were prepared using the program PyMOL or ChimeraX.

### 2.2 Animals

The knock-in mouse line *Otof^D1841/1842A^* (i.e., *Otof^DDA^*) was generated by site-directed CRISPR-Cas9 mutagenesis. The corresponding verified mutated sequences were as follows:

(5′)gtggctgaagggccagcaggaggacaaacaggacacagatgtccactatcactccctcacgggggagggcaacttcaactggagatacctcttccccttcgactacctagcggccgaagagaagatcgttatgtccaaaaaggagtctatgttctcctgggatgagacggagtacaagatccctgcgcggctcaccctgcagatctgggacgctga**T**cacttctcggctg**CT**g**C**cttcctgg (3′)

The sequence shown covers bp 5426–5667 (Exon43) of GenBank Acc. No. NM_031875, *Mus musculus* otoferlin (Otof), transcript variant 2, mRNA. The antisense KI-specific primer site is underlined, the D1841/1842A missense mutation is marked in red, and the silent mutation is marked in blue.

The successful insertion of *Otof^D1841/1842A^* was confirmed by genotyping according to standard PCR methods using DNA isolated from tail biopsies (using the Genomic DNA Isolation Kit for Tissue and Cells according to the manufacturer’s protocol; Nexttec, Hilgertshausen, Germany). For quality control, all samples were analyzed on a 1% agarose gel. PCR products were analyzed by fragment analyses on a 3730XL-DNA-Analyzer (Applied Biosystems, now Life Technologies, Darmstadt, Germany). Genotyping primers were synthesized in-house, and sequence and fragment sizes are as follows:

**Table 1:** 

**Primer name**	**Sequence**
37087	5'-CCAGCAGGAGGACA AACAGG-3'
37089	5'-TGCACCCAGGA AGTCGT-3'
37090	5′-CGAATG CACCCAGGAAGG CAG-3'

*Otof^D1841/1842A^*
37087_37089_235bp = OTODDA_WT_235
37087_37090_239bp = OTODDA_KI_239

Mice of either sex, aged between 2 weeks and 11 months, were used for experiments. Mice were kept in individually ventilated cages with environmental enrichment (cardboard rolls and wood wool) on a 12/12 h day/night cycle in the animal facility of the Max Planck Institute for Multidisciplinary Sciences. All experiments complied with national animal care guidelines and were approved by the University of Göttingen Board for Animal Welfare and the Animal Welfare Office of the State of Lower Saxony (AZ 19/3133 and AZ 19/3134).

### 2.3 Immunohistochemistry and confocal microscopy

Mice (2-week-old and 4-week-old) were deeply anesthetized with CO_2_ and sacrificed by decapitation for immediate dissection of the cochleae in ice-cold PBS. Fixation was performed by perfusing the cochlea with 4% formaldehyde (in PBS) for 60 min on ice, while for the labeling of synapses, a shorter fixation of 30 min was performed. The organs of Corti were dissected and washed briefly in PBS at room temperature. Blocking and permeabilization of the tissue were performed with goat serum dilution buffer (GSDB: 16% normal goat serum, 450 mM NaCl, 0.3% Triton X100, 20 mM phosphate buffer, pH ~7.4) for 1 h at room temperature. Samples were then incubated with primary antibodies (diluted in GSDB) overnight at 4°C and were washed three times for 10 min in wash buffer (450 mM NaCl, 0.3% Triton X 100, 20 mM phosphate buffer, pH ~7.4). This was followed by incubation with secondary antibodies (diluted in GSDB) for 1 h in a light-protected wet chamber. Finally, the samples were washed three times for 10 min in wash buffer before mounting onto glass slides with a drop of fluorescence mounting medium (Mowiol 4-88, Carl Roth, Karlsruhe, Germany) and covered with thin glass coverslips. Images were acquired in the confocal mode using an Abberior Instruments Expert Line STED microscope (Abberior Instruments GmbH, Göttingen, Germany). We employed lasers at 488, 561, and 633 nm for excitation. In the study, 1.4 NA 100X or 0.8 NA 20X oil immersion objectives were used. Confocal stacks were acquired using Imspector Software (Abberior Instruments GmbH, Göttingen, Germany; pixel size = 70 × 70 nm in xy, 200 nm in z). The acquired images were z-projected with NIH ImageJ software and adjusted for brightness and contrast. The organs of Corti from both mutant mice and corresponding heterozygote or WT mice were always processed in parallel using identical staining protocols, laser excitation powers, and microscope settings. Images were acquired and analyzed without *a priori* knowledge of the genotype.

**Table 2:** 

**Antibody**	**Host species**	**Company**	**Dilution**	**Identifier**
**Primary antibody**				
Anti-N-otoferlin	Mouse (monoclonal IgG1)	Abcam	1:300	ab53223
Anti-C-otoferlin (1215)	Rabbit		1:300	
Anti-parva lbumin	Chicken (polyclonal)	Synaptic Systems	1:300	195006
Anti-ctbp2 (ribeye)	Mouse (monoclonal IgG1)	BD Biosciences	1:300	612044
Anti-homer-l	Rabbit (polyclonal)	Synaptic Systems	1:500	160002
Anti-Vglut3	Rabbit (polyclonal)	Synaptic Systems	1:300	135203
Anti-GM130	Mouse (monoclonal IgG1)	BD Biosciences	1:200	610822
**Secondary antibody**				
STAR580 conjugated anti-mouse		Abberior	1:200	2-0002-005-1
STAR635p conjugated anti-rabbit		Abberior	1:200	2-0012-007-2
Alexa Fluor 488 conjugated anti-chicken		Invitrogen	1:200	A11039
Alexa Fluor 647 conjugated anti-rabbit		Invitrogen	1:200	A21244
Alexa Fluor 488 conjugated anti-rabbit		Invitrogen	1:200	A11008

### 2.4 Recordings of ABRs and otoacoustic emission

Recordings of ABRs and distortion product otoacoustic emission (DPOAE) in mice were performed largely as previously described (Jing et al., 2013). Mice were anesthetized by i.p. injection of a combination of ketamine (125 mg/kg) and xylazine (2.5 mg/kg). In all *in vivo* experiments, the core temperature was constantly maintained at 37°C using a heat blanket (Hugo Sachs Elektronik–Harvard Apparatus) or a custom-designed heat plate. To record ABRs, signals between subcutaneous needle electrodes at the vertex and mastoid were amplified 50,000 times, bandpass filtered between 400 and 4,000 Hz (Neuroamp), and averaged at least 2 × 1,300 times for each stimulus type using TDT System II and BioSig software (TDT). Stimuli included tone bursts of varying frequencies at up to 80 dB and clicks at up to 120 dB. DPOAE was recorded using Tucker Davis Technologies (TDT) System III and custom MATLAB software, TDT EC1 speakers, a Sennheiser MKE-2 microphone, and a Terratec DMX Fire or UAC zoom-2 microphone preamplifier. All stimuli were calibrated using a quarter-inch Bruel and Kjaer D 4039 microphone.

### 2.5 Patch clamp

Apical turns of the organs of Corti from 2-week-old mice were isolated in an ice-cold HEPES Hank’s solution containing (in mM) 5.26 KCl, 141.7 NaCl, 0.5 MgSO_4_.7H_2_O, 10 HEPES (4-(2-hydroxyethyl)-1-piperazineethane-sulfonic acid), 1 MgCl_2_, 11.1 D-glucose, and 3.42 L-glutamine; the pH was adjusted to approximately 7.2 and osmolality was ~300 mOsm/kg. The recording chamber was perfused with modified Ringer’s solution containing (in mM) 2.8 KCl, 111 NaCl, 35 TEA-Cl (tetraethylammonium chloride), 10 HEPES, 1 CsCl, 1 MgCl_2_, 11.1 D-glucose, and 2 CaCl_2_; the pH was adjusted to approximately 7.2 and osmolality was ~300 mOsm/kg. The tissue was cleaned to make the IHCs accessible for patch clamp by removing the tectorial membrane and neighboring cells. The clean exposed basolateral surface of IHCs was patch-clamped in perforated patch configuration, as described previously (Moser and Beutner, 2000), using an EPC-10 amplifier (HEKA Electronics, Germany) controlled by *Patchmaster* software at room temperature. The pipette solution contained (in mM) 137 Cs-gluconate, 10 TEA-Cl, 10 4-aminopyridine, 10 HEPES, 1 MgCl_2_, and 300 μg/mL of amphotericin B; the pH was adjusted to 7.2 using HCl and osmolality was ~290 mOsm/kg. Cells were kept at a holding potential of −87 mV. All voltages were corrected for liquid junction potential (17 mV) offline. Currents were leak-corrected using a p/10 protocol. Recordings were discarded when the leak current exceeded −50 pA, series resistance exceeded 30 MΩ, or Ca^2+^ current rundown exceeded 25%. Current–voltage relationships (IVs) were recorded once the access resistance dropped below 30 MΩ, by applying increasing 10 ms long step-depolarization pulses of voltage ranging from −87 mV to 65 mV, in steps of 5 mV. Exocytosis measurements were performed by measuring increments in membrane capacitance (∆C_m_) using the Lindau–Neher technique (Lindau and Neher, 1988). ∆C_m_ was recorded by stimulating the cells at the potential for maximal Ca^2+^ influx (−17 mV) for variable durations. Successive stimuli were acquired at an interval of 10–90 s. Each protocol was sequentially applied two to three times and only IHCs with reproducible measurements were included. For analysis, capacitance traces were averaged over 400 ms before and after the depolarization [skipping the first 60 ms after the end of depolarization to avoid the impact of non-exocytic C_m_ changes (Neef et al., 2007)]. The traces were subjected to 5 or 10 pass binomial smoothing using Igor Pro 6 (WaveMetrics Inc., Lake Oswego, United States) for display. All recordings were performed in the same sequence for better comparability with previous data.

### 2.6 Statistical analysis

Data were mainly presented as box and whisker plots presenting median, lower/upper quartiles, and 10–90th percentiles with individual data points overlaid or bar plots with mean ± SEM. Data were analyzed using Excel and Igor Pro 6 and 7 (WaveMetrics Inc.). Using Igor Pro, the normality of data was assessed with the Jarque–Bera test or the Wald–Wolfowitz test, and equality of variances in normally distributed data was assessed with the F-test. Differences between the two groups were evaluated for significant differences using the two-tailed unpaired Student’s *t*-test, or, when not normally distributed and/or variance was unequal, the unpaired two-tailed Mann–Whitney–Wilcoxon test was used. Non-significant differences between samples are indicated as *n.s.*, and significant differences are indicated as ^*^p < 0.05, ^**^p < 0.01, and ^***^p < 0.001.

## 3 Results

### 3.1 Generation of *Otof^DDA/DDA^* mice by CRISPR/Cas9 editing

The structure of otoferlin predicted by AlphaFold2 (Figure 1B) suggests that the C_2_F domain top loops are close to the transmembrane domain, which might be relevant for the ER targeting of otoferlin. A human missense mutation affecting the transmembrane domain impairs the transmembrane domain recognition complex subunit of 49 kDa (TRC40)-dependent ER targeting the tail-anchored protein otoferlin (Vogl et al., 2016). Here, using CRISPR/Cas9 editing, we substituted two conserved aspartate residues thought to contribute to Ca^2+^ binding by the C_2_F-top loops by alanine residues (D1841/1842A, *Otof^DDA/DDA^*) in mice (Figures 1C,D). Superovulated C57BL/6 N females were mated with C57BL/6 N males, and fertilized eggs were collected. In-house prepared CRISPR reagents (hCAS9_mRNA, sgRNAs, and either dsDNA or long oligonucleotides were used as repair templates containing the desired mutation) or preformed Cas9_sgRNA RNP complexes were microinjected into the pronucleus and the cytoplasm of zygotes at the pronuclear stage using an Eppendorf Femtojet (see Section 2 for further details). Note that all nucleotide-based CRISPR-Cas9 reagents (sgRNAs and hCAS9_mRNA) were used as RNA molecules and were not plasmid-coded. In this way, we intended to reduce the likelihood of off-target effects as RNA-based reagents are only short-lived (Doench et al., 2016; Tycko et al., 2019) in contrast to plasmid-coded reagents. Successful insertion of *Otof^D1841/1842A^* (*Otof^DDA/DDA^*) was confirmed by genotyping (see Materials and Methods). *Otof^DDA/DDA^* mice were born at the Mendelian ratio and did not show obvious abnormalities upon routine observation.

### 3.2 Altered subcellular otoferlin distribution and reduced basolateral otoferlin levels in IHCs

**Figure 2:**
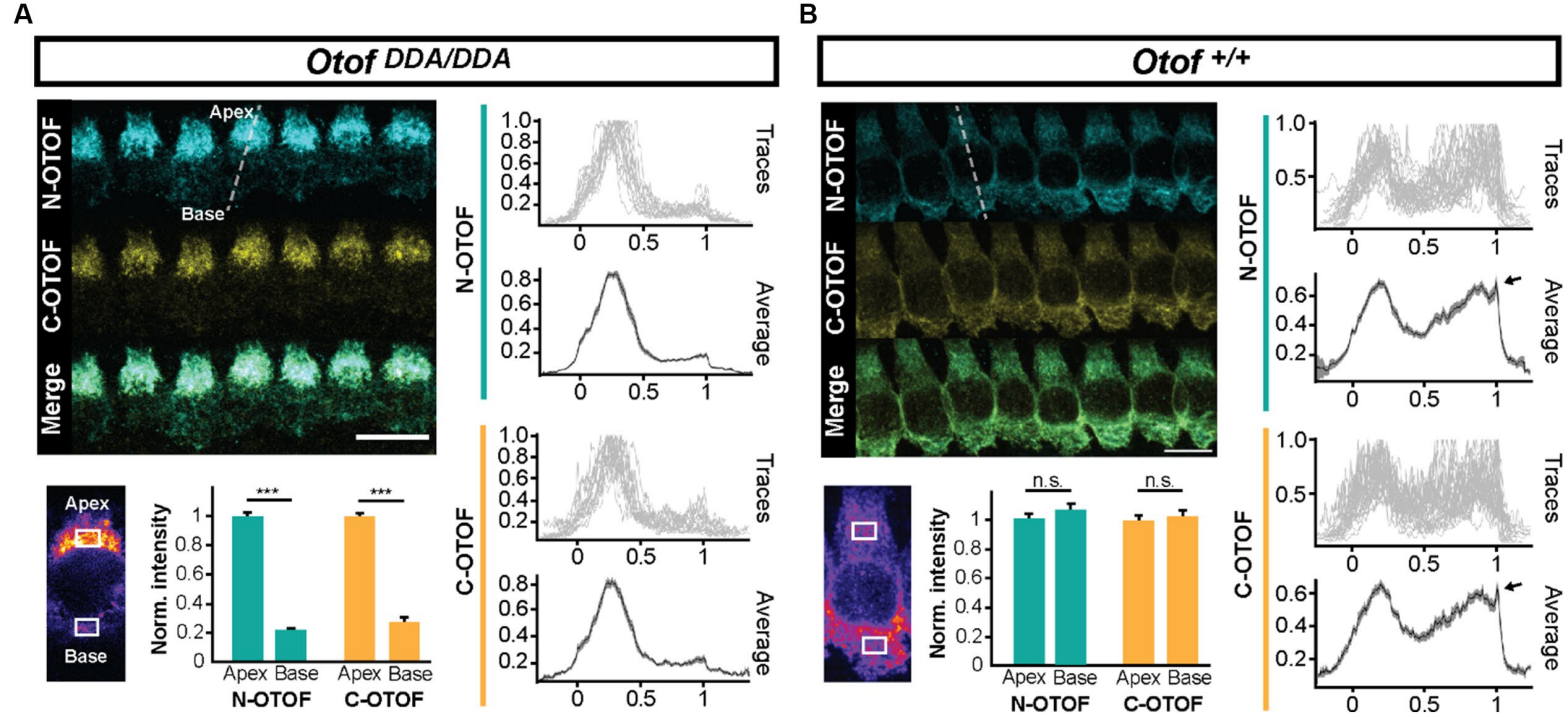
IHCs of *Otof^DDA/DDA^* mice exhibit an abnormal subcellular otoferlin distribution. **(A)** Line profile analysis (indicated as a white dashed line) shows that otoferlin levels in *Otof^DDA/DDA^* IHCs are considerably higher in the apex than in the base. **(B)** Line profile analysis shows that otoferlin levels in *Otof^+/+^* IHCs are largely comparable between the apex and the base. The fluorescence peak at the basal edge (arrowheads in **B**) corresponds to otoferlin expression in the plasma membrane.

**Figure 3:**
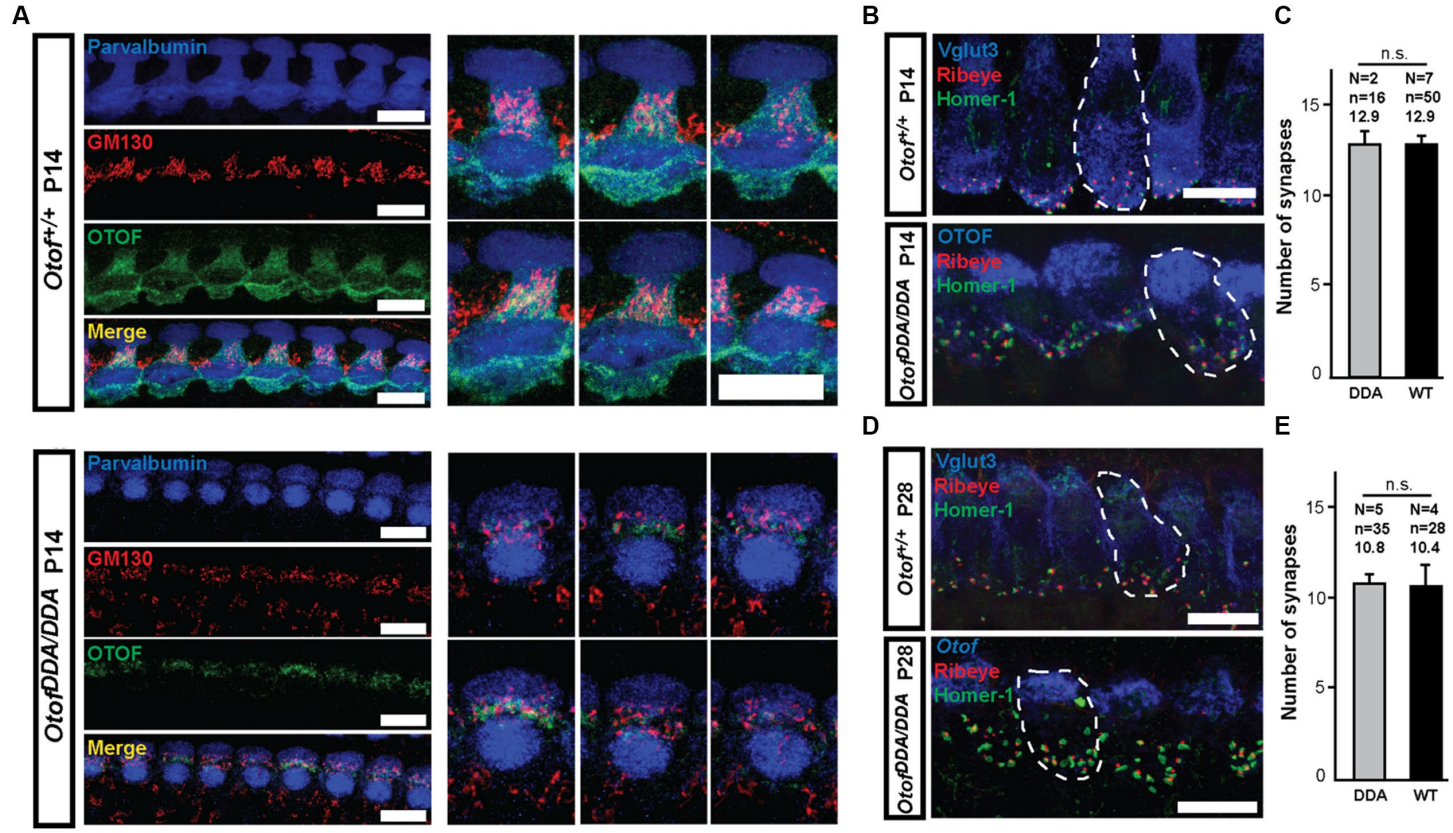
DDA-otoferlin does not seem to accumulate in the Golgi apparatus—normal number of ribbon synapses in *Otof^DDA/DDA^* IHCs. **(A)** Maximum intensity projections of confocal stacks of *Otof^DDA/DDA^* **(A, bottom)** and *Otof^+/+^* **(A, top)** IHCs from P14 mice following co-immunolabeling for parvalbumin (blue), Golgi marker GM130 (red), and otoferlin (green). **(B)** Maximum-intensity projections of confocal stacks of P14 mouse *Otof^DDA/DDA^* (bottom) and *Otof^+/+^* (top) IHCs following immunolabeling for Vglut3 (blue), Ribeye (red), and Homer-1 (green). Pairs of juxtaposed red and green immunofluorescence were counted as ribbon synapses. Scale bar, 10 μm. **(C)** The numbers of ribbon synapses per IHC are similar between *Otof^DDA/DDA^* (gray) and *Otof^+/+^* (black) mice at P14 (mean ± SEM). **(D)** Maximum-intensity projections of confocal stacks of P28 mouse IHCs of different genotypes following immunolabeling for Vglut3 (blue), Ribeye (red), and Homer-1 (green). Pairs of juxtaposed red and green immunofluorescence were counted as ribbon synapses. Scale bar, 10 μm. **(E)** The numbers of ribbon synapses per IHC were similar between *Otof^DDA/DDA^* mice (gray) and *Otof^+/+^* (black) mice at P28.

Otoferlin is known to distribute broadly in IHCs (Roux et al., 2006; Pangrsic et al., 2010; Revelo et al., 2014) from the apex of the IHC where the Golgi is localized (Siegel and Brownell, 1986; Vranceanu et al., 2012; Revelo et al., 2014) to the basal pole that contains the afferent ribbon synapses. Semiquantitative analysis of otoferlin immunofluorescence (antibodies to C- and N-terminal epitopes) showed an altered subcellular distribution of otoferlin in *Otof^DDA/DDA^* IHCs (Figure 2). Line profile analysis revealed a stark apex to the base gradient of otoferlin immunofluorescence in IHCs. Preliminary comparison to *Otof^DDA/+^* IHCs indicated an upregulation of apical otoferlin levels (~doubling) and a ~ 40% reduction of basal otoferlin levels in *Otof^DDA/DDA^* IHCs (Supplementary Figure 1). We considered the possibility of an impaired membrane targeting of the mutant otoferlin because of the accumulation of otoferlin in the IHC apex. Hence, we performed further immunohistochemistry for otoferlin and the Golgi marker GM130 (Figure 3A). However, we did not find indication of an accumulation of otoferlin in the Golgi and assume that the excess signal in the apex arises from non-targeted otoferlin as the AlphaFold2 prediction indicates an interaction of the C_2_F-top loops with the transmembrane domain that might be relevant for ER targeting. We note that nuclear staining by the IHC context marker parvalbumin has been described in previous studies (Pangršič et al., 2015) and that differences in cytosolic and nuclear parvalbumin immunofluorescence likely reflect different loss of cytosolic parvalbumin during permeabilization. Future studies might consider immunostaining for other compartments such as the endoplasmic reticulum. Importantly, the basolateral otoferlin levels of *Otof^DDA/DDA^* IHCs (60%) exceed those of *Otof^D1767G/D1767G^* (25%; Pangrsic et al., 2010) IHCs that show intact phasic Ca^2+^ exocytosis and of *Otof^I515T/I515T^* IHCs with intact phasic and better maintained sustained exocytosis than in *Otof^D1767G/D1767G^* IHCs (35%; Strenzke et al., 2016).

Next, we evaluated the IHC-SGN connectivity and found ribbon synapses, identified as pairs of juxtaposed presynaptic Ribeye/CtBP2 and postsynaptic Homer-1 immunofluorescence spots, to be present in normal number per *Otof^DDA/DDA^* IHCs at the end of the second and fourth postnatal week (Figures 3B–E). This contrasts findings in other *Otof* mutants that show a loss of synapses of ~50% (*Otof* KO; Roux et al., 2006; Stalmann et al., 2021) mice and ~20% (*Otof^D1767G/D1767G^*; Pangrsic et al., 2010), which indicates a partial functionality of DDA-otoferlin.

### 3.3 Auditory synaptopathy in *Otof^DDA/DDA^* mice

**Figure 4:**
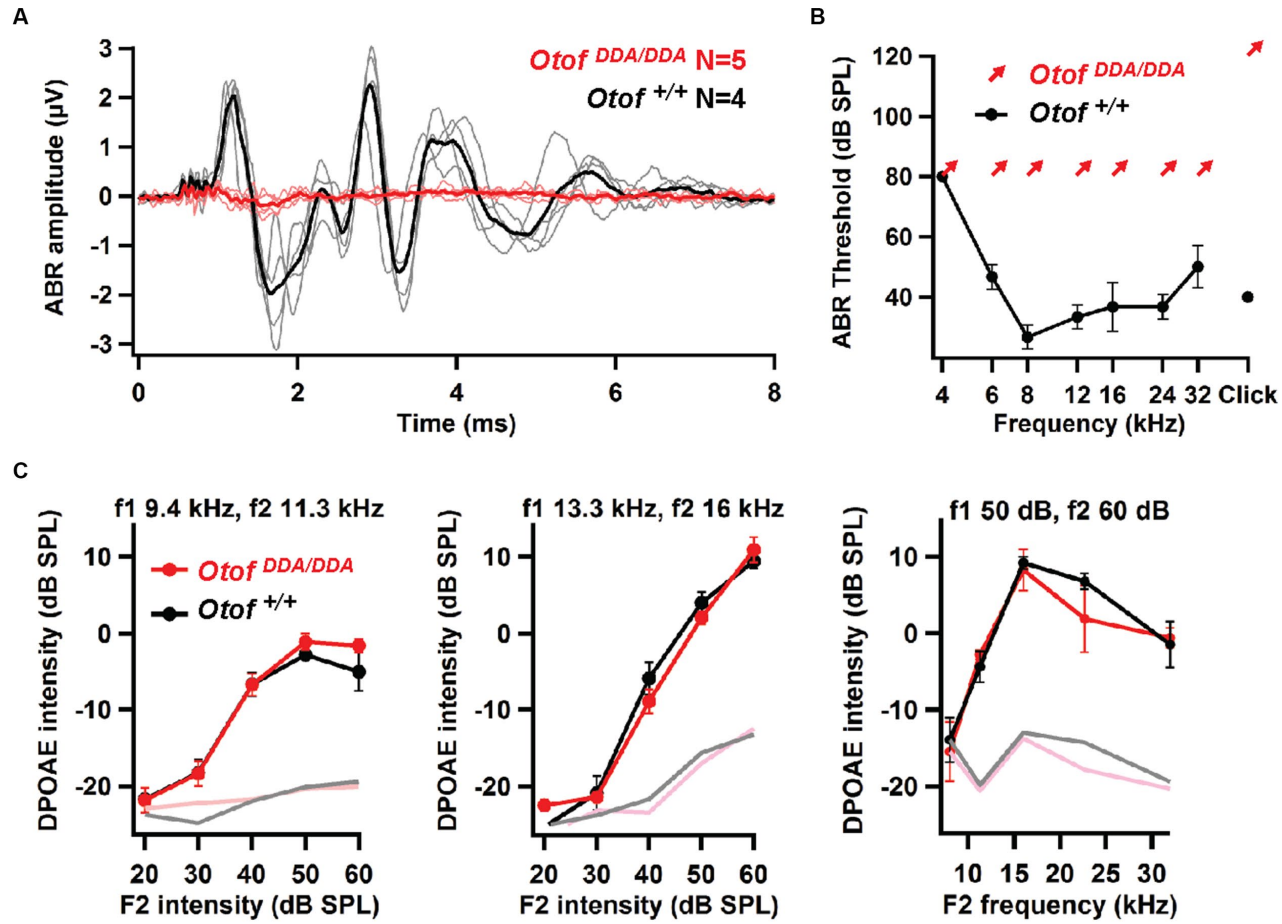
*Otof^DDA/DDA^* mice lack auditory brainstem responses despite intact otoacoustic emissions. **(A)** No ABRs were elicited by 100-dB click stimuli in 5-week-old *Otof^DDA/DDA^* mice (individuals: pink, grand mean: red), whereas sizable ABRs were seen in *Otof^+/+^* controls (individuals: gray, grand mean: black). **(B)** *Otof^+/+^* mice had normal ABR thresholds, whereas no ABRs were detected in *Otof^DDA/DDA^* mice up to our maximal loudspeaker output of 80 dB SPL for tone bursts and 120 dB (peak-equivalent) for clicks, arrows indicated non-detectable thresholds at the given sound pressure levels. **(C)** Left and middle: DPOAE growth function for two different pairs of stimulating primary tones: low frequency (left) and mid-frequency (middle); right: DP-gram (amplitude for different pairs of primary tones for different frequency pairs). Robust DPOAE with normal amplitudes for primary tones of different frequencies in *Otof^DDA/DDA^* mice indicates normal OHC function across the cochlear frequency range.

Recordings of auditory brainstem responses (ABRs; see Materials and Methods) indicated a loss of synchronized activation of spiral ganglion neurons (SGNs) and propagated neural activity along the early auditory pathway of *Otof^DDA/DDA^* mice (Figures 4A,B). Despite sizable summating potentials, reflecting intact hair cell receptor potentials, we did not detect SGN compound action potential (ABR wave I) or brainstem responses (waves II–IV) in 5-week-old homozygous *Otof^DDA/DDA^* mice which were obvious in *Otof^+/+^* mice. Next, we recorded otoacoustic emissions to test for cochlear amplification mediated by outer hair cells (Ashmore, 2008). Distortion product otoacoustic emissions (DPOAEs, see Section 2) were observed across the cochlear frequency range indicating intact cochlear amplification by outer hair cells (OHCs; Figure 4C). The lack of ABRs despite intact OHC function signifies auditory synaptopathy or neuropathy (Moser and Starr, 2016), which is also found in another C_2_F mouse mutant (*Pachanga*: *Otof^D1767G/D1767G^*). Given the role of otoferlin in hair cell exocytosis, the deafness of *Otof^DDA/DDA^* mice most likely reflects a synaptopathy, which we further investigated by patch-clamp recordings from IHCs.

### 3.4 Largely abolished exocytosis despite intact Ca^2+^ currents in IHCs of *Otof^DDA/DDA^* mice

**Figure 5:**
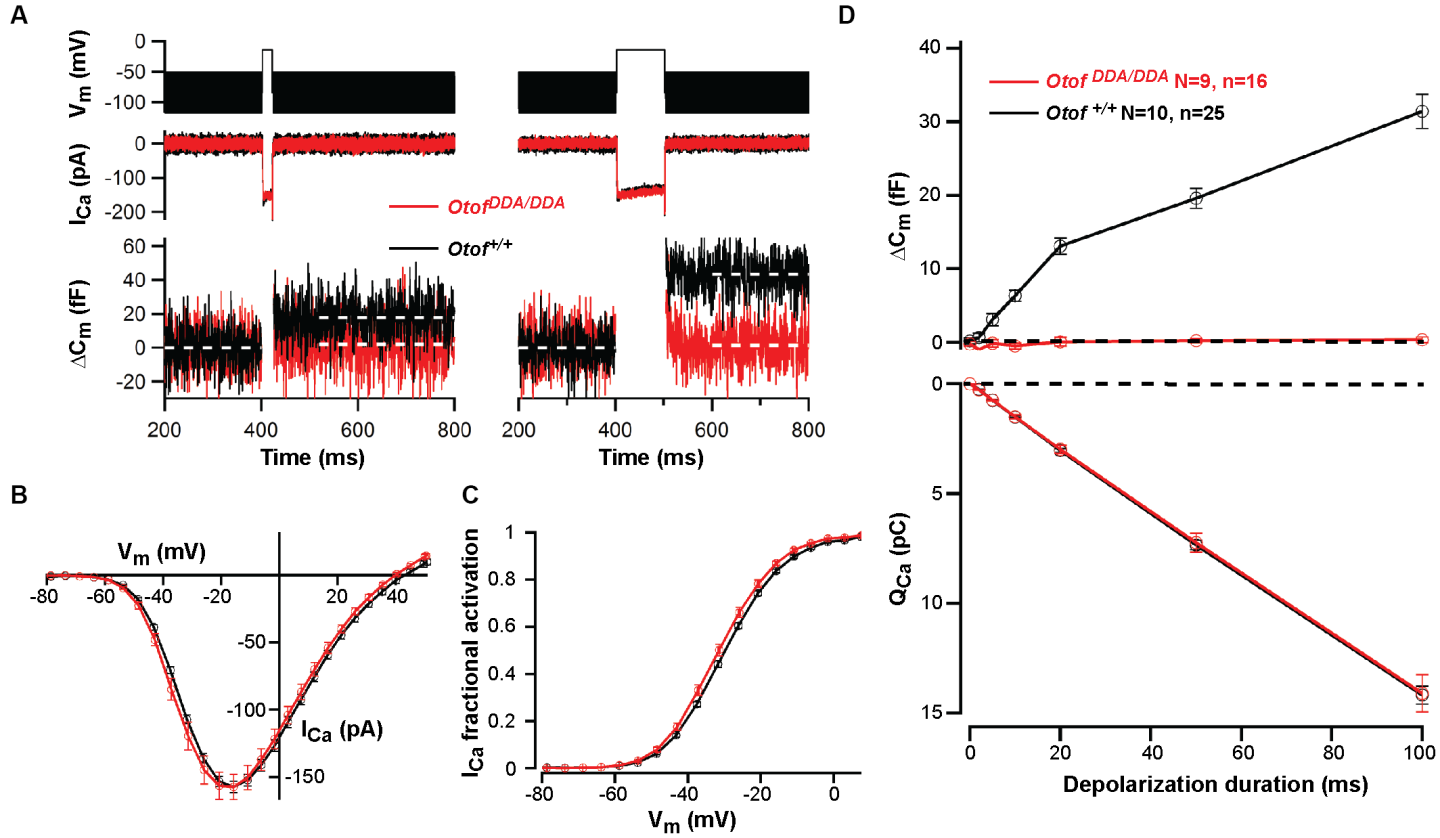
Abolished exocytosis in *Otof^DDA/DDA^* IHCs despite normal Ca^2+^ influx. **(A)** Representative Ca^2+^ currents (middle panel) and changes in membrane capacitance (ΔC_m_; lower panel) of *Otof^DDA/DDA^* (red) and control *Otof^+/+^* IHCs (black) in response to depolarization (upper panel, left, 20 ms; right, 100 ms) to the voltage where maximum Ca^2+^ currents were elicited (−14 mV). Lack of exocytic ΔC_m_ in *Otof^DDA/DDA^* IHCs. **(B,C)** No significant difference in voltage-dependent Ca^2+^ currents **(B)** and fractional activation of Ca^2+^ channels **(C)** between *Otof^DDA/DDA^* and *Otof^+/+^* mice (mean ± SEM). **(D)** Exocytic ΔC_m_ (top) is present in IHCs of *Otof^+/+^* mice but lacking in IHCs of *Otof^DDA/DDA^* mice (P14-18 mice, *N* = number of mice, *n* = number IHCs, mean ± SEM) despite identical Ca^2+^ current integrals (bottom, corresponding to ΔC_m_).

In order to further address the effects of the C_2_F mutations on presynaptic IHC function, we performed perforated-patch recordings of voltage-gated Ca^2+^ influx and exocytic membrane capacitance changes (ΔC_m_) from IHCs of *Otof^DDA/DDA^* mice in the third postnatal week [postnatal day 14–18 (P14-18)]. Voltage-gated Ca^2+^ influx showed normal amplitudes and voltage-dependence of activation (Figures 5A–C), which differs from reports such as on *Otof^D1767G/D1767G^* IHCs, which showed a 20% reduction of IHC Ca^2+^ influx (Pangrsic et al., 2010). This was attributed to a mild loss of IHC synapses because the presynaptic Ca^2+^ signals were comparable in *Otof^D1767G/D1767G^* IHCs and their controls. However, despite intact Ca^2+^ influx *Otof^DDA/DDA^* IHCs largely lacked Ca^2+-^ triggered exocytosis (Figure 5D), again contrasting *Otof^D1767G/D1767G^* IHCs that showed intact exocytosis of the readily releasable pool of synaptic vesicles (Pangrsic et al., 2010). In fact, the ΔC_m_ of *Otof^DDA/DDA^* IHC was comparable to that of *Otof^−/−^* IHCs (Roux et al., 2006; Pangrsic et al., 2010; Reisinger et al., 2011). We consider this to reflect the impact of impaired Ca^2+^ binding to DDA-otoferlin. Resting membrane capacitance did not differ significantly among IHCs of the different genotypes, which together with the normal number of ribbon synapses and Ca^2+^ currents suggests that the global physiology of *Otof^D1841/1842A^* IHCs is unaltered.

## 4 Discussion

In the present study, we used mouse mutagenesis to examine the role of the most C-terminal C_2_F domain of otoferlin in IHC presynaptic function and hearing. Genetic perturbation focused on the top loops that are involved in Ca^2+^ binding by substituting putative Ca^2+^ binding aspartate residues with alanines (D1841/1842A) in CRISPR/Cas9 generated in *Otof^DDA/DDA^* knock-in mice. In line with an auditory synaptopathy, we could not detect ABRs despite intact cochlear amplification by OHCs, as assessed by otoacoustic emissions. Abolished synaptic sound encoding was due to a lack of Ca^2+^ influx triggered exocytosis despite substantial remaining otoferlin expression and an intact number of IHC synapses. Specifically, we found a reduction to 60% for the basolateral synaptic pole, but higher than normal otoferlin immunofluorescence levels in the apical compartment of IHCs that harbors the Golgi apparatus and other membrane organelles that carry otoferlin (e.g., Revelo et al., 2014). We did not find evidence for the mutant otoferlin to accumulate in the trans-Golgi network. Different from other *Otof* mouse mutants, we found the afferent IHC synapses to be maintained. We conclude that the C-terminal C_2_F domain is critical for otoferlin function. Aside from the likely role in Ca^2+^ triggered membrane SV fusion, the Ca^2+^ bound pocket of the C_2_F domain might contribute to membrane targeting of otoferlin.

Our overall conclusion from analyzing the novel *Otof* mouse mutant is that the C_2_F domain contributes to Ca^2+^ sensing for SV fusion. However, the mutations caused additional alterations such as reduced otoferlin levels and/or disturbed subcellular otoferlin distribution that need to be considered carefully. Previous analysis of mouse mutants with reduced levels of otoferlin, be it due to *Otof* mutation (Pangrsic et al., 2010; Jung et al., 2015b; Strenzke et al., 2016) or disruption of interacting proteins (Jung et al., 2015b; Vogl et al., 2016), showed that Ca^2+^ triggered SV fusion is robust down to at least 25% of WT levels (*Otof^D1767G/D1767G^*), while the rate of SV replenishment is more sensitive to reduced otoferlin levels. Basolateral otoferlin levels in *Otof^DDA/DDA^* IHCs amounted to ~60% which exceeds otoferlin levels in *Otof^D1767G/D1767G^* and *Otof^I515T/I515T^* IHCs that showed intact SV fusion. Hence, the near complete lack of Ca^2+^ influx-triggered SV exocytosis of *Otof^DDA/DDA^* IHCs cannot be merely due to the reduced otoferlin levels. Instead, it likely reflects an impairment of Ca^2+^ sensing for SV fusion although other possibilities cannot be ruled out unequivocally. For example, aberrant otoferlin interaction with the Ca_V_1.3 Ca^2+^ channel complex (e.g., via C_2_F; Ramakrishnan et al., 2009; Hams et al., 2017) could loosen the otherwise tight spatial coupling of SV release sites to Ca^2+^ channels. We speculate that the selective reduction of basolateral otoferlin levels reflects impaired membrane targeting of the basolateral plasma membrane. The Ca^2+^ binding top loops of the C_2_F domain are close to the transmembrane domain (Figure 1B), and their alteration might affect the efficient targeting of the membrane, which relies on the TRC40-dependent pathway for targeting tail-anchored proteins (Vogl et al., 2016). Future analysis of purified DDA-otoferlin, including TRC-40 dependent ER-targeting, will help to test this hypothesis. This will also help to identify the potential effects of the mutations on the interaction of otoferlin with Ca_V_1.3 Ca^2+^ channels (Ramakrishnan et al., 2009; Hams et al., 2017).

Finally, combinatorial mutagenesis experiments targeting Ca^2+^ binding sites of several C_2_ domains should be pursued to dissect the Ca^2+^ sensing functions of otoferlin in SV fusion and replenishment (Pangrsic et al., 2010; Michalski et al., 2017). Although CRISPR/Cas9 gene editing has massively expedited the generation of mouse mutants, it will be important, e.g., for rapid functional analyses, to optimize viral gene transfer for efficient expression of selected otoferlin mutants using *Otof^−/−^* IHCs. Expression of full-length (Akil et al., 2019; Al-Moyed et al., 2019; Rankovic et al., 2021) and partial deletion constructs of otoferlin (mini-otoferlins; Tertrais et al., 2019) via AAV-mediated gene transfer partially restores function in *Otof^−/−^* IHCs. However, achieving WT levels of otoferlin, especially of mutant variants, will likely remain difficult to achieve. As mentioned above, SV fusion in IHCs tolerates lower levels of otoferlin than SV replenishment, restoration of which seems to be required for restoring auditory signaling (Al-Moyed et al., 2019). Interestingly, while expression of full-length otoferlin in *Otof^−/−^* IHCs led to partial restoration of ABRs in all three studies published (Akil et al., 2019; Al-Moyed et al., 2019; Rankovic et al., 2021), ABRs were not observed upon expression of mini-otoferlins (Tertrais et al., 2019). Unfortunately, the autaptic neural culture system, a workhorse in molecular synapse physiology, or chromaffin cells do not provide a workaround here as their synaptotagmin cannot be substituted by transgenic otoferlin (Reisinger et al., 2011). Analysis of afferent hair cell synapses in the lateral line organ of zebrafish provides a valuable option (Trapani and Nicolson, 2011; Sebe et al., 2017; Sheets et al., 2017; Wong et al., 2019) and has been successfully employed for studying otoferlin function (Chatterjee et al., 2015).

## Data availability statement

The datasets presented in this study can be found in online repositories. The names of the repository/repositories and accession number(s) can be found in the article/Supplementary material.

## Ethics statement

The animal study was approved by the University of Göttingen Board for Animal Welfare and the Animal Welfare Office of the State of Lower Saxony (AZ 19/3133 and AZ 19/3134). The study was conducted in accordance with the local legislation and institutional requirements.

## Author contributions

HC: Conceptualization, Data curation, Formal analysis, Investigation, Methodology, Software, Validation, Visualization, Writing – original draft. QF: Formal analysis, Investigation, Writing – review & editing. FB: Resources, Validation, Writing – review & editing. NB: Resources, Supervision, Writing – review & editing. TM: Conceptualization, Funding acquisition, Resources, Supervision, Validation, Writing – original draft.
