# Supplementary figures and images for: Probing the role of the C2F domain of otoferlin
Source: Front Mol Neurosci. 2023 Dec 12;16:1299509. doi: 10.3389/fnmol.2023.1299509 (PMC10751786; doi:10.3389/fnmol.2023.1299509)

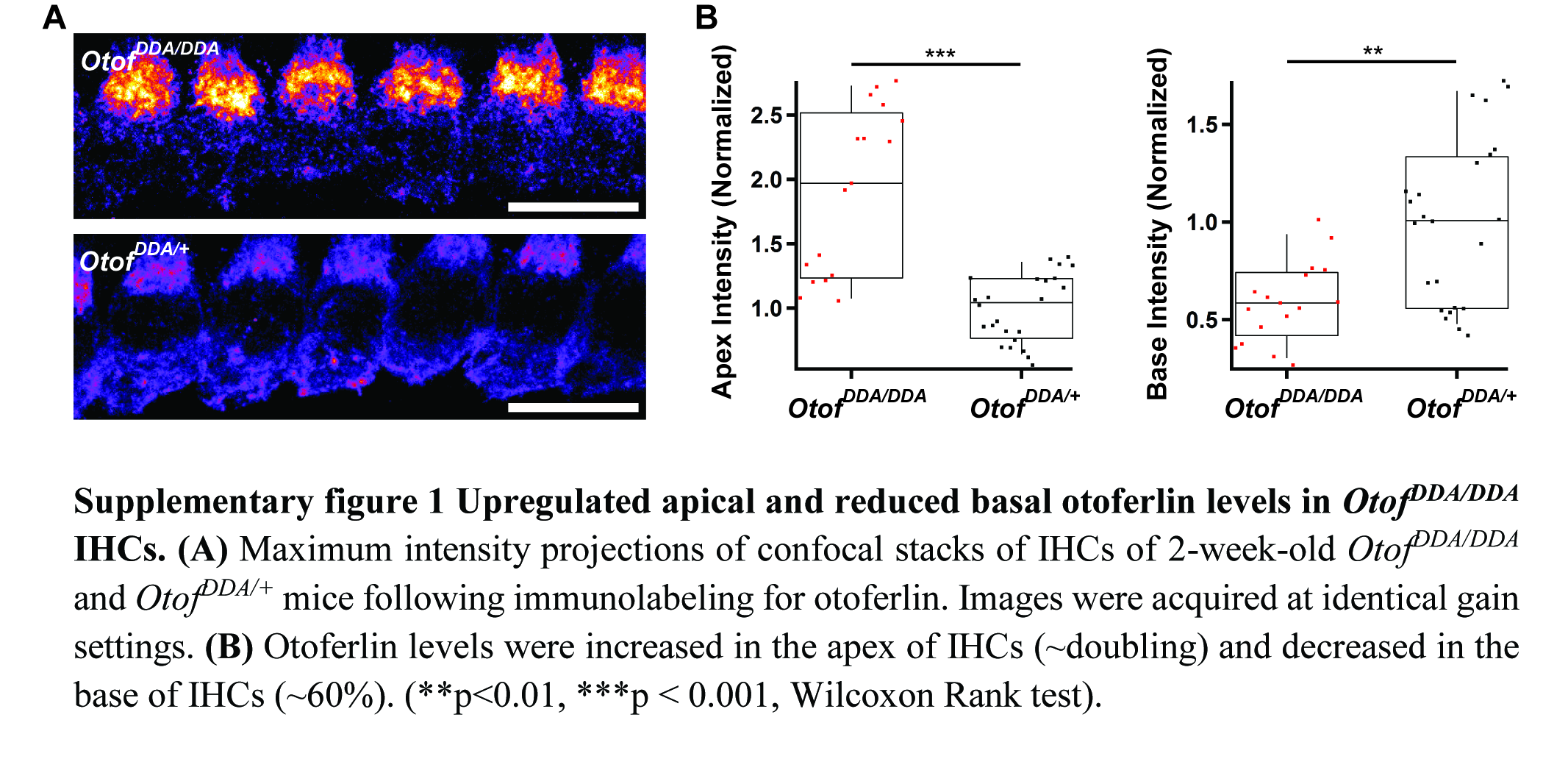

Supplement: Supplementary file 1 [file Image_1.TIF]
